# Cellulose-Based Materials and Their Application in Lithium–Sulfur Batteries

**DOI:** 10.3390/polym17020164

**Published:** 2025-01-10

**Authors:** Muriel Zampieri, Guillermina Tommasone, Luciana Morel, Guillermina Leticia Luque

**Affiliations:** 1 Facultad de Matemática, Astronomía, Física y Computación, Universidad Nacional de Córdoba (UNC), Córdoba 5000, Argentina; muriel.zampieri@unc.edu.ar; 2 Instituto de Física Enrique Gaviola (IFEG), Consejo Nacional de Investigaciones Científicas y Técnicas (CONICET), Córdoba 5000, Argentina; 3 Instituto de Investigaciones en Físico-Química de Córdoba (INFIQC), Consejo Nacional de Investigaciones Científicas y Técnicas (CONICET), Córdoba 5000, Argentina; gtommasone@unc.edu.ar (G.T.); luciana.morel@unc.edu.ar (L.M.); 4 Departamento de Química Teórica y Computacional, Facultad de Ciencias Químicas, Universidad Nacional de Córdoba (UNC), Córdoba 5000, Argentina

**Keywords:** cellulose-based materials, lithium–sulfur batteries, lithium metal anode, separator, cathode

## Abstract

Lithium–sulfur (Li-S) batteries are promising candidates for next-generation energy storage due to their high energy density, cost-effectiveness, and environmental friendliness. However, their commercialization is hindered by challenges, such as the polysulfide shuttle effect, lithium dendrite growth, and low electrical conductivity of sulfur cathodes. Cellulose, a natural, renewable, and versatile biopolymer, has emerged as a multifunctional material to address these issues. In anode protection, cellulose-based composites and coatings mitigate dendrite formation and improve lithium-ion diffusion, extending cycle life and enhancing safety. As separators, cellulose materials exhibit high ionic conductivity, thermal stability, and excellent wettability, effectively suppressing the polysulfide shuttle effect and maintaining electrolyte stability. For the cathode, cellulose-derived carbon frameworks and binders improve sulfur loading, conductivity, and active material retention, resulting in higher energy density and cycling stability. This review highlights the diverse roles of cellulose in Li-S batteries, emphasizing its potential to enable sustainable and high-performance energy storage. The integration of cellulose into Li-S systems not only enhances electrochemical performance but also aligns with the goals of green energy technologies. Further advancements in cellulose processing and functionalization could pave the way for its broader application in next-generation battery systems.

## 1. Introduction

Every year, there is an increase in energy demand due to population growth, accompanied by a demand for higher life quality, as well as economic development. Many of the new renewable energy resources such as sun and wind are intermittent, bringing a concomitant need for some form of energy storage during their energy production to be used when needed [1].

**Figure 1:**
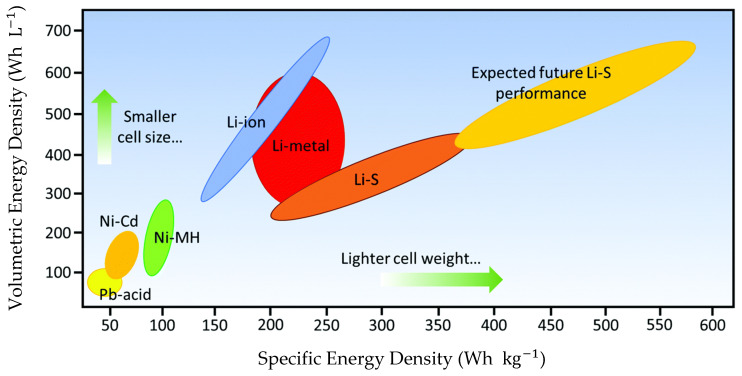
Ragone plot comparing different rechargeable battery technologies as a function of specific and volumetric energy densities. Printed with permission of [2].

In this context, lithium-based batteries play a fundamental role, as they can store more energy per volume and weight than most rechargeable batteries and possess higher working voltage, more significant energy density, and prolonged cycle life (see Figure 1) [2]. Today’s most widely used lithium batteries are lithium-ion batteries (LIBs), used in electric vehicles and small electric devices (tools, cell phones, laptops, among others). Nevertheless, this technology, based on the insertion and extraction between active anode and cathode materials, has reached its theoretical limit of 265 W h kg^−1^ [3].

The need for new greener materials with higher specific capacity and energy density gave rise to new technologies, such as lithium–sulfur (Li-S) batteries that present higher theoretical energy density of up to 2600 W h kg^−1^, although from the practical standpoint, the energy density of a packaged Li–S battery could potentially be up to about 600 W h kg^−1^ [4]. Another advantage of these batteries is their low cost and abundance of sulfur. Moreover, sulfur cathodes are more environmentally friendly when compared to metal transition materials used as cathode in Li-ion batteries [5].

In the search for renewable, cost-effective materials, natural environmentally friendly polymers, such as cellulose-based ones, have gained attention due to their abundance, availability, renewability, biodegradability, thermal and chemical stability, and high versatility that allow their application in many fields [6,7,8,9,10,11]. Cellulose advantages can be exploited for energy storage. It presents high mechanical strength and flexibility, making it suitable for manufacturing battery separators, electrodes, or surface modification. Its high thermal stability is a fundamental property that guarantees battery safety. Hydroxyl groups present on the cellulose make it particularly interesting when dealing with the shuttle effect in lithium–sulfur batteries, as they interact with polysulfides through electrostatic interactions. This will be discussed further in this review.

This review will present the working principle of Li-S batteries, their advantages, and challenges in their application. Then, cellulose-based materials will be discussed, along with their properties and the advantages of their use as lithium anode protection, separators, and as part of cathode material within Li-S batteries. Finally, conclusions, future directions, and challenges in applying cellulose-based materials in Li-S battery technology are discussed.

## 2. Working Principle of Li-S Batteries

**Figure 2:**
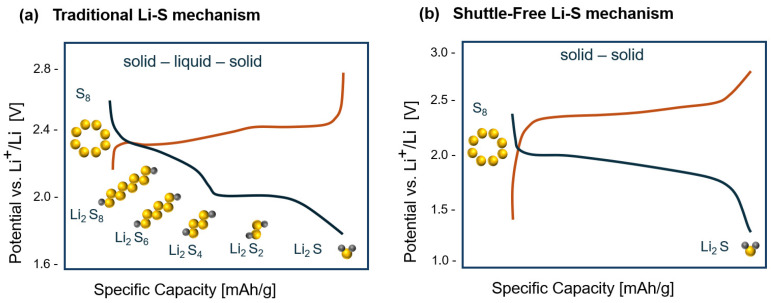
Schematic of operating principles of LSBs for (**a**) solid–liquid–solid conversion and (**b**) solid–solid conversion. Blue and red lines for discharge and charge process respectively. Reproduced with permission from ref. [15].

As an electrochemical storage device, electrical energy is stored within the sulfur cathodes of Li–S batteries. A Li–S cell typically starts with a discharge process since sulfur is in a charged state. Instead of the intercalation mechanism that takes place in Li-ion batteries, traditional Li-S cells present a redox conversion reaction with multielectron transfer and undergo a solid–liquid–solid transition as the reaction takes place. During discharge, lithium metal oxidizes, producing lithium ions that move through the electrolyte and electrons through the external circuit producing current, and solid S_8_ reduces accepting Li^+^ to form long-chain soluble polysulfides (hereafter referred to as “LiPs”) (Li_2_S_x_, 6 < x ≤ 8) that is seen as a “plateau” at approximately 2.4 V (solid to liquid conversion) [5]. These long-chain polysulfides are soluble in the commonly used liquid organic electrolytes, producing what is generally known as the “shuttle effect”. LiPs diffuse from the cathode through the separator to the anode, reacting with it and leading to the loss of the active material and a decrease in the reversible capacity [12]. The second plateau around 2.1 V corresponds to a reduction in soluble short-chain polysulfides to solid Li_2_S_2_, and the final slope to the reaction from Li_2_S_2_ to Li_2_S that are deposited on the surface of the cathode and anode (Figure 2) [13,14]. During the charging process, the reverse reaction takes place, from Li_2_S to S_8_.

In contrast, a solid-solid electrochemical conversion between sulfur and Li_2_S occurs when using solid electrolytes (SSEs) or, in some circumstances, in liquid electrolytes [15,16,17,18]. In this case, all solid-state lithium–sulfur batteries undergo an overall redox reaction, which can be represented as S_8_ + 16Li ↔ 8 Li_2_S being around 2.15 V (vs. Li/Li+), with no formation of polysulfides, preventing the shuttle effect.

**Figure 3:**
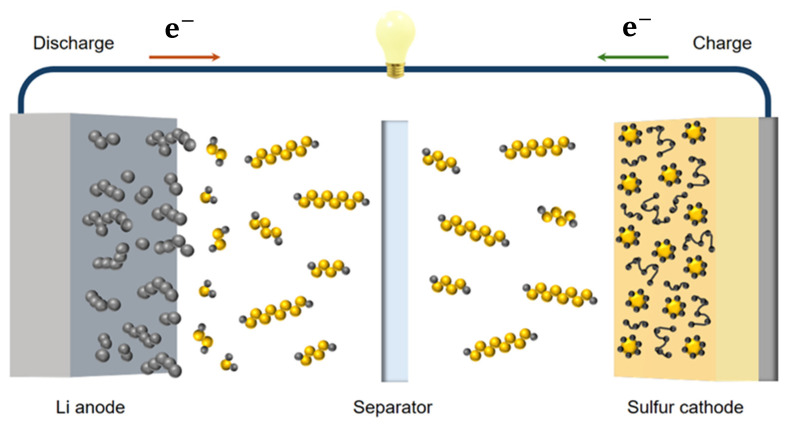
Schematic representation of the components within a Li–S cell with the arrows indicating charge/discharge processes.

Traditional lithium–sulfur batteries are composed of metallic lithium as an anode, a separator to prevent contact between the two electrodes, and a cathode material where the active material is sulfur in an organic-based electrolyte (Figure 3). These components will be the main focus of this review; however, solid-state electrolytes will also be discussed.

Starting with the anode, lithium metal is one of the most promising materials due to its high theoretical specific capacity of 3860 mAh g^−1^ (ten-times higher than graphite conventionally used as anode in LIBs) and low electrochemical potential of −3.04 V vs. the standard hydrogen electrode [19]. The working mechanism for the Li anode is the deposition and dissolution of Li^+^ ions on its surface instead of intercalation/deintercalation that occurs on the graphite anode during the charge/discharge process. During the repetitive charge/discharge cycle, the continuous deposition and irregular separation of lithium induce an uncontrollable growth of lithium dendrites, which not only breaks the interfacial electrolyte (SEI) film and leads to the generation of “dead Li” but also continues to consume the electrolyte, which results in low coulombic efficiency (CE) and bad cyclability. Dendrites also induce safety hazards (such as internal short circuits and combustion/explosion) as they can penetrate through the separator and form micro short circuits between the positive and negative electrodes, causing serious safety issues, including fire and even explosions [20,21,22].

The separator is a porous polymeric membrane sandwiched between the positive and negative electrodes in a cell. It prevents physical and electrical contact between the electrodes while permitting ion transport [23,24]. Although a separator is an electrochemically inactive element of a battery, characteristics of separators, such as porosity, pore size, mechanical strength, and thermal stability, influence ion transport, cycle life, performance, and safety [25,26]. Thus, the separator represents one of the key components in lithium batteries. Porous separators face multiple challenges in the electrochemical cell; its properties affect the safety of the cells. The occurrence of mechanical/electrical/thermal abuse scenarios during battery operation can physically damage the separator, leading to physical contact between the electrodes and internal short circuits [23,24,25]. In the case of electrical abuse, repetitive charge/discharge processes can lead to lithium dendrite (Li-dendrite) growth, which can penetrate through the separator. In extreme cases, these failures may trigger fires or explosions [26,27,28]. Designing a separator membrane with ideal characteristics is a way to maximize the charge transport kinetics, mitigate separator failures, and prevent premature battery failures. In Li-S batteries, the separator also plays an important role in the redox reaction of LiPs and the shuttling effect.

Solid-state electrolytes can replace separators in batteries, as they also serve to avoid electronic contact and prevent short circuits. These materials improve battery safety by eliminating the need for liquid electrolytes, making the commercialization of lithium anode batteries more viable, inhibiting dendrite formation [17,29]. As mentioned earlier, all-solid-state Li-S batteries avoid the shuttle effect as there is no dissolution of LiPs.

Considering the cathode active material, sulfur is one of the most abundant elements in the Earth’s crust; it is environmentally friendly and cost-effective and offers a high theoretical capacity of 1675 mA h g^−1^, which is an order of magnitude higher than those of transition-metal oxide cathodes [3,5,30]. The high capacity is attributed to the conversion reaction of sulfur to form lithium sulfide (Li_2_S), which is reversibly incorporated by two electrons per sulfur atom as opposed to one electron per metal-ion in cathodes that use transition-metal oxides. Nevertheless, Li-S batteries face the problem of sulfur’s poor ionic and electronic conductivity and its low-order reduction products (Li_2_S and Li_2_S_2_), increasing the battery’s internal resistance and, thus, the polarization. Further, other problems these batteries experience are the 80% volume change during reduction and expansion and the central problem of the shuttling effect produced by the dissolution of long-chain polysulfides (Li_2_S_x_, with 3 ≤ x ≤ 6) in the electrolyte, leading to loss of the active material and low coulombic efficiency [3,4,5].

## 3. Cellulose’s Main Characteristics

**Figure 4:**
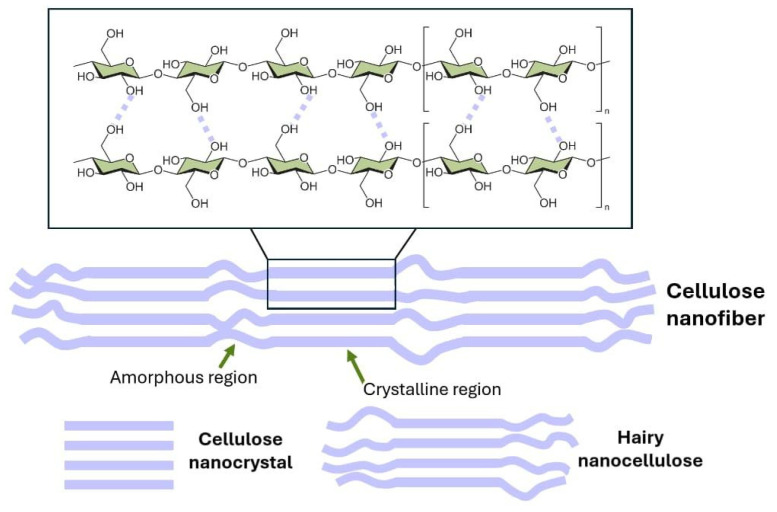
Schematic representation of crystalline and amorphous domains in cellulose structures.

Cellulose is a linear polymer that consists of repeated β(1–4)-D-glucose units. It is the most abundant biopolymer on Earth, present in plants, some algae and bacteria, and a few specific animals like tunicates. Cellulose chains form a parallel assembly through van der Waals forces and hydrogen bonds caused by the hydroxyl groups present [31,32,33]. This arrangement leads to the formation of cellulose fibrils, which can aggregate to form microfibrils. These consist of highly organized crystalline regions due to the hydrogen bonding and amorphous regions (Figure 4) [33,34].

Cellulose can be classified according to its size between micro- and nanocellulose [35]. Nanocellulose can be subdivided into cellulose nanofibrils (CNFs), cellulose nanocrystals (CNC), hairy cellulose nanocrystals (HCNC), and bacterial cellulose (BC) (Figure 4). CNFs usually have 5–50 nm diameter and several microns in length; they possess crystalline and amorphous regions. Nanofibrils are flexible and have high mechanical strength, which is helpful for film forming and flexible electrode fabrications. They also possess a large specific surface area due to their high aspect ratio [36,37]. CNFs can be obtained through mechanical methods, often with biological or chemical pretreatment to facilitate the process and reduce energy costs [38].

CNCs are typically produced by acid hydrolysis; they are elongated rod-like particles that contain the crystalline region of the cellulose chain with few amorphous segments. Due to the higher crystallinity, they also have a higher rigidity when compared to CNFs. Their dimensions go from 3 to 50 nm in width and 50 to 500 nm in length [39,40].

Generally, nanocellulose comes in the form of CNF or CNC; however, a newer form of nanocellulose, hairy nanocellulose, is possible to obtain from a controlled oxidation reaction. HNCs are cellulose nanocrystals with amorphous ends attached to the rods [41,42]. These “hairs” can be functionalized in a facile manner, leading to four different subcategories according to the functionalization: electrostatically stabilized nanocrystalline cellulose (ENCC), sterically stabilized nanocrystalline cellulose (SNCC), bifunctional nanocrystalline cellulose (BNCC) and cationic nanocrystalline cellulose (CNCC) [43].

Some bacteria can synthesize cellulose with a bottom-up approach via fermentation, with a higher degree of crystallinity, polymerization, and purity than cellulose obtained through other methods. Bacteria release protofibrils from the cell wall, which assemble into nanofibrils. This leads to the formation of a membrane on the surface of the culture medium. Culturing conditions can be tuned to modify the obtained nanocellulose [44]. BC fibers assemble in a cross-linked network structure, showing better tensile strength, flexibility, and water absorption [45,46]. This structure is maintained after carbonization, making BC ideal as flexible electrodes [47].

Cellulose is insoluble in water and most solvents, hindering its processing. In order to solubilize cellulose, multiple cellulose derivatives have been obtained by chemical modification of the hydroxyl groups. Some of the most available derivatives include cellulose acetate, carboxymethyl cellulose, methylcellulose, and cellulose sulfate, among others. Facile functionalization of the cellulose can enhance its properties, such as ionic conductivity, thermal stability, or mechanical strength [48]. Another common form of cellulose is regenerated cellulose, obtained from dissolving and then precipitating cellulose with a nonsolvent. Some of the most common solvents used are ionic liquids [49,50,51], N-methylmorpholine N-oxide (NMMO) [52,53,54], LiCl/Dimethylacetamide [55,56,57], alkali/urea aqueous solution [58,59,60], or deep eutectic solvents (DESs) [61,62,63]. Cellulose has four polymorph types characterized by different hydrogen bonding sites between the chains, called I, II, III, and IV. Natural cellulose is found in the form of cellulose I, and its dissolution and regeneration can transform it into cellulose II [64,65].

## 4. Application of Cellulose-Based Materials in Li-S Batteries

**Figure 5:**
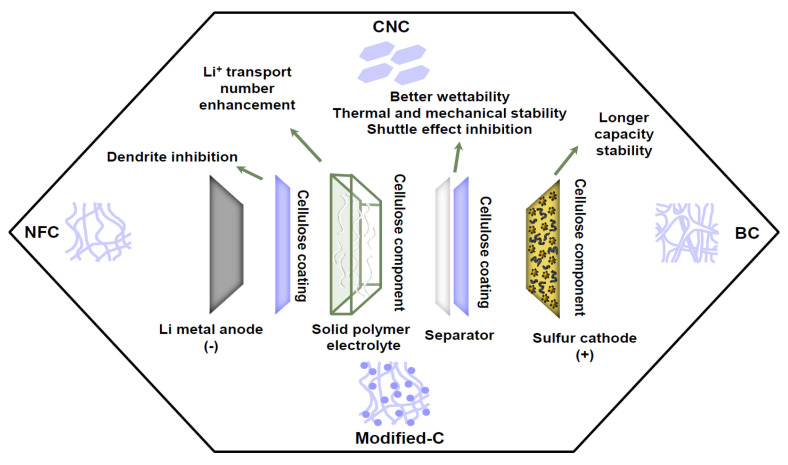
Application of cellulose-based materials and its advantages on their application as components of different parts of Li-S batteries.

Many of the unique combinations of structural, chemical, and electrochemical properties of cellulose-based materials can improve some of the aforementioned problems related to lithium–sulfur batteries. Cellulose possesses multiple benefits, such as great versatility (tunable properties, functionalization potential), abundance, biodegradable nature, robustness (mechanical strength, thermal and chemical resistance), high porosity and surface area, high wettability that allows for higher ionic conductivity, polysulfide trapping capability, and scalability in the production (Figure 5). All these make cellulose a compelling material to use in all components of Li-S batteries, as will be discussed in this section.

### 4.1. Cellulose for Lithium Metal Protection

As mentioned in the Introduction, dendrite formation due to the inhomogeneous deposition of lithium ions during the charge and discharge processes is a recurring problem in the development of lithium-metal-based batteries. This leads to a short life cycle, capacity loss, and security issues.

There are various approaches to address this problem. Even though some of the presented results are applied to other types of cathodes different from sulfur, the same strategies can be applied when using sulfur as a cathode material.

Solid-state electrolytes (SSEs) appear as a promising alternative to inhibit dendrite formation due to their higher mechanical strength and better chemical stability over liquid-state electrolytes [66,67,68]. In Li-S, SSEs are an effective method not only to minimize Li dendrite formation but also to prevent the dissolution of LiPs [69,70]. Among the diverse biomasses used as materials for the creation of SSE [71], cellulose, in all of its forms, is portrayed as an attractive compound in the design of SSEs because of its favorable mechanical qualities, abundance, and environmental friendliness. Despite their potential, SSEs must overcome obstacles like lowering production costs, increasing interface stability, and optimizing ionic conductivity. However, this field of study is developing quickly, and SSEs are thought to be a major step towards safer, more effective, and longer-lasting batteries. Cellulose-based solid polymer electrolytes (CSPEs) are particularly attractive and present great promise because of their multiple merits, including abundant reserves, abundant polar groups, high compatibility with lithium chemical and mechanical stability, and high flexibility [72]. Regrettably, effective lithium-ion transport in SPEs is compromised by the high stiffness needed to suppress dendrites. Prado-Martinez et al. [73] used cellulose to create a composite SPE based on comprised soft poly(ethylene oxide co-epichlorohydrin) (EO-co-EPI) with cellulose nanofibers (CNFs), which combines high ionic conductivity from the copolymer with great stiffness from the CNFs. As the lithium electrolyte salt, LiTFSI was employed. To characterize the CSPE, the authors performed dynamic mechanical analysis (DMA) and electrochemical impedance spectroscopy (EIS) measurements, where they found a higher ionic conductivity (6 × 10^−5^ S cm−1) and storage modulus (64 MPa) in the cellulose-modified SPE. They then performed electrochemical studies to evaluate the performance of the SPE. Cyclic voltammetry experiments were carried out on Li/Cu cells and exhibited a stability window between 0 V and 4 V. To prove dendrite growth suppression, galvanostatic cycling experiments on Li/Li cells were performed, and the author found a longer life cycle in CNF-modified SPE.

Methyl 2-hydroxyethyl cellulose (HEMC) was utilized by Wu et al. [74] as an organic filler component of CPSE to strengthen a PEO-based polymer electrolyte. Composite polymer electrolytes are compressed by compositing inorganic particles into solid polymer electrolytes, summing up the advantages of polymer electrolytes with enhanced ionic conductivity and mechanical properties conferred by the inorganic particles [75], enhancing the electrochemical and physicochemical stability. These authors [71] found PEO-LiTFSI-20HEMC, with a proportion of 20 wt% HEMC, to be optimal, which was added to a homogeneous solution of mixed PEO and LiTFSI with a molar ratio of 16:1 (EO:Li) in anhydrous acetonitrile. Compared to pure PEO-based systems, utilizing cellulose HEMC gives polymer electrolytes greater interface compatibility with the Li anode, higher Li-ion transference number (0.467), and better mechanical strength (Young’s modulus of 46.95 MPa and tensile strength of 3.26 MPa). Benefits of anode dendrite suppression and conductivity were found in the modified CSPE. Li/PEO16-LiTFSI-20HEMC/LiFePO_4_ cells exhibit stable long-term cycling (~120 mAh g^−1^ at 1 C for 300 cycles) and high-rate performance (80 mAh g^−1^ at 2 C).

**Figure 6:**
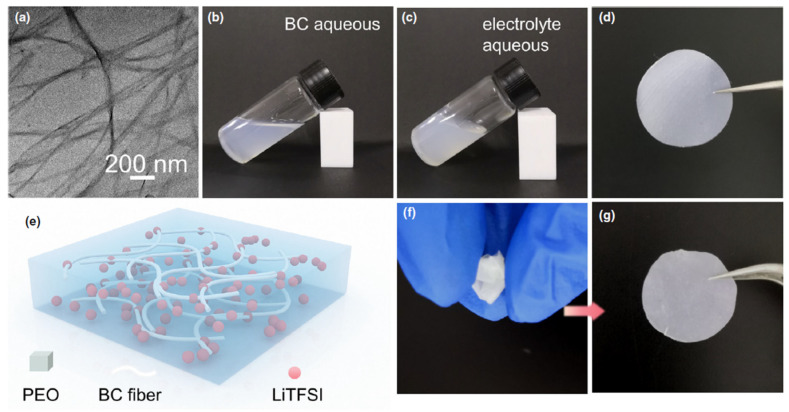
Morphology analysis of solid polymer electrolyte. (**a**) TEM image of BC. (**b**) The BC solution. (**c**) The solution of BC composite electrolyte. (**d**) The photograph of PEO/LiTFSI/BC CSPE film. (**e**) Schematic illustration of the composition of the BC-PEO CSPE. (**f**,**g**) Photographs of PEO/LiTFSI/BC CSPE film after mechanical perturbation. Reproduced with permission from [73].

Li et al. [76] prepared a high-performance CSPE using BC as a polymer filler incorporated with Poly(ethylene oxide) (PEO) in a LiTFSI-based electrolyte. The composite solid polymer electrolytes were prepared by aqueous mixing in water. They achieved well-dispersed nanofibers in the electrolyte PEO-LiTFSI-BC CSPE exhibiting considerable tensile strength and a low degree of crystallinity. The random arrangement of BC nanofibers is presented in Figure 6a, where average diameter sizes of 50–100 nm can be observed. Figure 6b–d show photographs of the BC suspension in water, POE, and LiTFSI mixed in the aqueous dispersion to form a uniform electrolyte solution, and finally, the solid polymer electrolyte film where the solution is poured onto the disk. Figure 6e displays the detailed schematic structure of the PEO-LiTFSI-BC (CSPE), where the BC nanofiber, LiTFSI, and PEO are represented, respectively, by a milky white fiber, a pink ball, and a blue block (continuous phase). After folding and bending, the PEO/LiTFSI/BC CSPE film could return to its former shape (Figure 6f,g).

**Figure 7:**
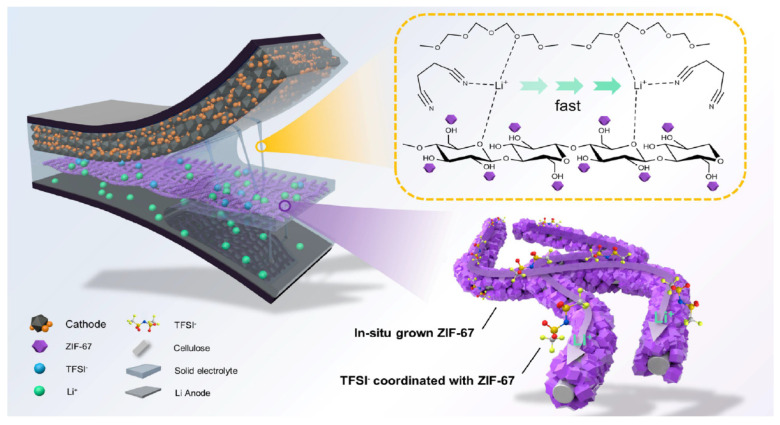
Schematic structure of ZIF-67@CF/PEO-SN CPEs. Reproduced with permission from [77].

The electrochemical characterization in a Li/Li symmetrical cell and Li/LiFePO_4_ showed an increase in the lithium-ion transfer number, being 0.57 for the PEO-LiTFSI-BC CSPE compared to 0.409 for the unmodified PEO-LiTFSI SPE. Similarly, they found a higher tensile strength in the PEO-LiTFSI-BC CSPE (4.43 MPa) versus the unmodified PEO-LiTFSI SPE (1.34 MPa). Additionally, they discovered that all solid-state metallic Li batteries using PEO/LiTFSI/BC CSPE have a 600-cycle lifespan, as opposed to 50 cycles for PEO/LiTFSI SPE batteries. The PEO-LiTFSI-BC CSPE symmetrical Li battery remained stable after 1160 h of cycling. Song et al. [77] used commercial membrane cellulose fibers (CFs) as the skeleton of the CSPE along with a soft polymer electrolyte. The CSPE consisted of a succinonitrile (SN) plasticizer in addition to zeolitic imidazolate frameworks (ZIFs) grown in situ on the CF skeleton, all into PEO (named as CSPE as ZIF-67@CF-PEO-SN) acting synergically to enhance the physicochemical and electrochemical properties. The lithium electrolyte salt utilized in this work was LiTFSI. Figure 7 shows a diagram of the CSPE composition structure. The authors found that numerous open metal sites of Co^2+^ in ZIF-67 can bind with anions (TFSI^−^) in electrolyte-filled pore channels, increasing Li^+^ dissociation and mobility and establishing a fast and continuous Li^+^ conducting pathway exhibiting high ionic conductivity, transference number, great tensile strength, and better stability, diminishing the dendrite lithium formation. Furthermore, density functional theory (DFT) and COMSOL simulation were used to confirm the Li^+^ conduction mechanism in the CSPEs.

Polyurethane-cellulose acetate was also investigated for a CSPE by Wang et al. [78] in a blend with mono-hydroxysiloxane-capped polyethylene oxide (PU-CA). The authors investigated the compatibility of CSPE with different lithium salts, LiBOB, LiBF_4_, LiTFSI, and LiNO_3_. The authors found a conductivity maximum of 1.5 × 10^−5^ S/cm with the addition of LiTFSI and, according to them, the greater degree of dissociation of the alkali metal salts inside the polymer matrix is likely to cause this enhanced conductivity. Ionic conductivity rises as a result of this dissociation because there are more free lithium ions distributed throughout the polymer, enabling them to take part in ion transport more actively. Table 1 presents different CSPEs that use cellulose and are applied to lithium metal systems. Bacterial cellulose was also used as a BC/MOF composite for gel electrolytes by Fu et al. [79], who used two hierarchical mesoporous Zr-based MOFs, finding that the one with NH2-UiO-66 as a linker increases the Li+ transference number and the electrochemical stability, prolonging the cycle life. Gel electrolytes present the advantage over SPE of having better ionic conductivity and good interface compatibility; nevertheless, the redox kinetic conversion of sulfur leads to slow LiPs conversion, leading to the shuttle problem. Huang et al. [70] designed an asymmetric cellulose gel electrolyte and UiO66/black phosphorous coating layer that allowed them to simultaneously address the problem of lithium dendrite generations and shuttle of LiPs.

In liquid electrolyte systems, different dendrite inhibition methods were investigated using cellulose-based materials. One of many consists of a superficial cellulose-based barrier on top of the lithium anode acting as a shield [87,88] by arranging the nucleation sites and preventing the formation of dendrites. In the last few years, cellulose composites were used for the same purpose, in the anode protective layer composition. Chang et al. [89] used Kimwipe (KW) commercial paper and modified the Li electrode surface (as received without any modifications) as a protective layer. KWs are composed of a virgin wood fiber framework. The abundant polar functional groups from the cellulose fibers are the main inspiration to the authors, based on different studies where a 3D network with polar functional groups helped in an even Li-ion distribution and obtained a free dendrite surface. Li/LiCF_3_SO_3_-DOL:DME/Li symmetric cell modified and non-modified galvanostatic cycled at different current densities to directly prove the dendrite formation. The authors found that the KW modification prevents dendrite formation and transforms preexisting dendrites into a smooth layer. They attribute these results to the abundance of polar functional groups in the modifying material, increasing the possibilities for a uniform Li-ion deposition on the electrode surface, instead of gathering around the protrusions.

Hong et al. [90] used a superficial modification on a Li metal anode, composed of cellulose nanofibers (CNFs) and copper nanowires (CuNWs). The gradient conductivity (CG) host was used as a method for suppressing dendrite formation. It was composed of three layers, the first, right on top of the anode, consisting of CuNW and being highly conductive; the second conductive medium consisted of CuNW+CNF, and the last exposed at the electrolyte was made of CNF and SiO_2_ nanoparticles, and it had low conductivity. The authors characterized the material using SEM images, X-ray spectra, and electrical resistance measurements. To study the dendrite inhibition performance, they performed lithium plating and stripping on top of Cu foil, CuNW mesh, and CG host, finding dendrite formation on the first one, with deposited lithium located mostly on the top surface in the CuNW mesh and smooth and stable deposition on the CG host. The experimental observations were matched with the electrical field COMSOL calculations, where dendrite formation on the Cu foil exhibited a great amount of hot spots, therefore favoring its continuous formation. The CuNW mesh also presented hot spots for being highly conductive, and a homogenization of the local electric field was found in the case of the CG host. Electrochemical performance was analyzed for the modified surface by Li symmetric galvanostatic cycling and the full cell with NCM811 as the cathode material. The authors found that the gradient conductivity interface stabilizes Li-metal anodes exhibiting a stable cycling performance at high current densities and homogeneous Li deposition. Lastly, the authors attribute the even distribution of the Li-ion reaction to the polar groups of the CNFs.

**Figure 8:**
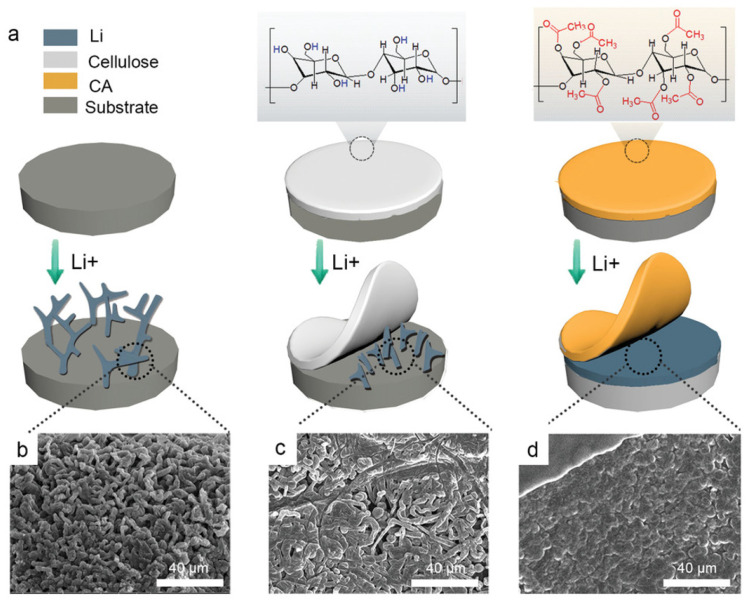
(**a**) Diagram of Li deposition on bare Cu, cellulose-Cu and CA-Cu electrodes. SEM images of Li deposition at 4 mAhcm^−2^. (**b**) Cu electrode, (**c**) cellulose-Cu electrode and (**d**) CA-Cu electrode. Reproduced with permission from [91].

Cellulose acetate (CA) was introduced as a protective coating on the Li metal anode by Chen et al. [91] in order to produce SEI with quick Li+ diffusion kinetics; ion-affiliative CAs with functional Li salts (LiN(CF_3_SO_2_)_2_ and LiTFSI) were synthesized. Continuous Li deposition was made on top of a Cu foil substrate to prove the dendrite inhibition formation. Figure 8a shows the deposition Li diagram in modified and non-modified substrates, according to morphology results from SEM images (b, c, and d). The electrochemical performance was analyzed through galvanostatic cycling of the Li symmetrical cell. The Li deposition morphology through SEM images of the anodes was studied after cycling. The authors found a way to improve the full cell electrochemical performance with the modification and demonstrated that functional ester groups and Li salts (studied by Cryo-TEM, EDS and XPS) were correlated with C=O strong adsorption on TFSI- via electrostatic contact. This enhances the charge transfer-promoted breakdown of LiTFSI, producing a rich LiF SEI.

**Figure 9:**
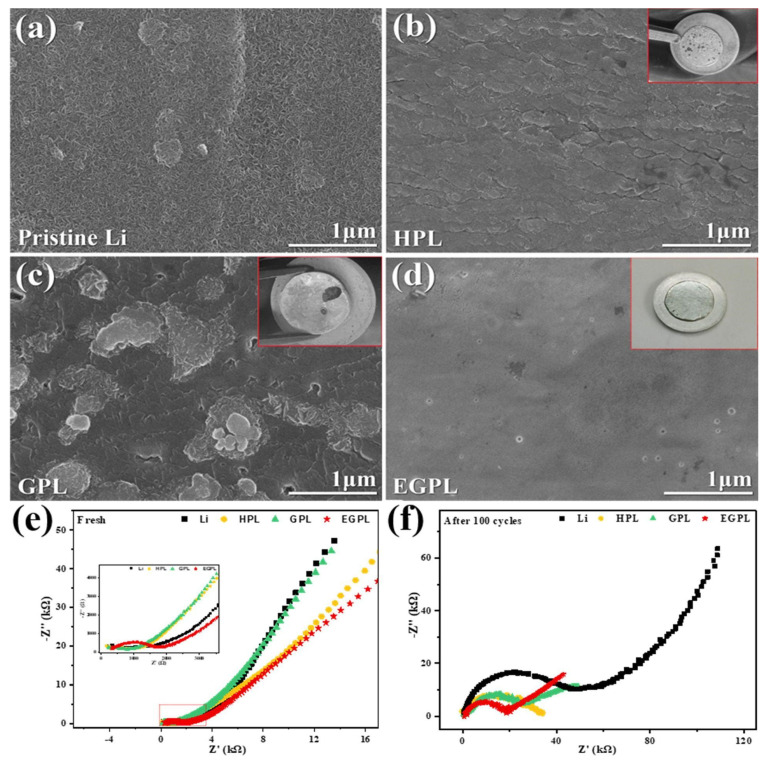
SEM images of Li deposition after 40 cycles at 0.2 C. (**a**) Pristine Li electrode, (**b**) HPL electrode, (**c**) GPL electrode and (**d**) GPL electrode. EIS spectra measurements (**e**) before and (**f**) after 100 cycles. Reproduced with permission from [92].

Wu et al. [92] used ethyl cellulose (EC) to create a composite based on EC, graphene oxide, and phosphoric acid. This artificial SEI was fabricated in situ on top of the Li anode surface by drop casting the modifying solution and then spinning the electrode. The authors of this work focused on designing an interlayer to improve both contact and interfacial stability as well as dendritic inhibition for solid-state LMB. The SEM images after 40 cycles of non-modified Li, EGLP, and the other two modifications using only phosphoric acid (HPL) and using graphene oxide plus phosphoric acid (GPL) are shown in Figure 9a–d, where major uniformity in the EGPL-modified anode can be seen, in contrast to the others, where flakes and dendrites can be observed. EIS spectra realized for the same samples before and after 100 cycles are also shown in Figure 9e,f, where it can be observed that the development of dendrites and impedance was consistently correlated; a rise in dendrite formation corresponds to an increase in cell impedance. For the initial cells, the impedances of HPL, GPL, and EGPL were substantially greater than pure Li due to the lithium metal surface coating. After 100 cycles, the EGPL cell exhibited a lower impedance than the GPL, HPL, and Li cells.

Finally, electrochemical measurements were carried out in full cells, varying the modified anode with pristine Li, GPL, HPL, and EGPL. The authors found that the introduction of the coatings improved the interphase stability, by efficiently preventing the formation of lithium dendrites, promoting uniform Li deposition, and enhancing the transport of lithium ions. When EC was added, the artificial layer became extremely compact and offered superior interfacial compatibility between the sulfide solid electrolyte and the lithium anode. As a result, the EGPL Li anode coat exhibited better rate and cycling capabilities as compared to bare Li anode batteries.

### 4.2. Cellulose for Separators

Cellulose-based separators or coatings on conventional polyolefins separators can greatly improve the performance of Li-S batteries. Conventional polyolefin separators suffer major drawbacks, such as poor wettability and low thermal stability [24]. Separators made with different materials or modifications of the polyolefin separators can improve these characteristics, as well as preventing the polysulfide shuttle effect and the growth or penetration of lithium dendrites.

**Figure 10:**
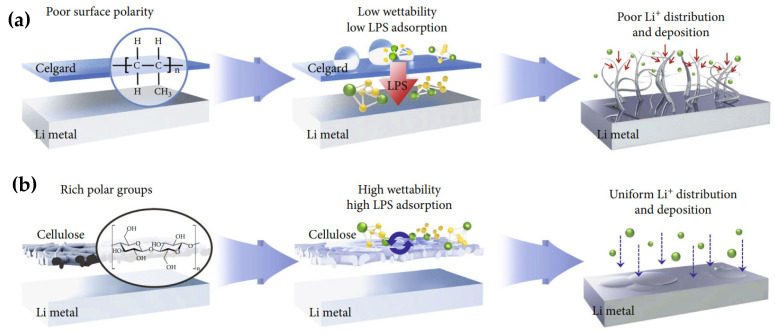
Schematic of the potential mechanisms through which the cellulose separators stabilize Li metal anode effects of the (**a**) Celgard and (**b**) cellulose separators on Li deposition. Reproduced with permission from [96].

Cellulose presents higher thermal stability than conventional polyolefin separators [93,94]. Moreover, cellulose increases the wettability and electrolyte uptake of the separators due to its polar functional groups [10]. These functional groups cause quicker absorption of the electrolyte and better storage ability of the separator. High electrolyte uptake and uniform pore structure lead to a better ionic conductivity, enhancing lithium-ion mobility and decreasing the internal resistance of the battery [94,95]. Multiple studies have shown a more homogeneous lithium deposition and inhibition of lithium dendrites when using cellulose. This comes from the combination of good wettability and uniform Li ion flux [96,97,98], as illustrated in Figure 10, and high mechanical strength of the separator [99,100,101].

Oxygen-containing functional groups interact with polysulfides, successfully diminishing the shuttle effect. Hydroxyl groups on the cellulose can lose their H^+^ ions, leading to a negatively charged surface [102,103]. Some authors explained the inhibition of the shuttle effect as adsorption of the polysulfides on the separator’s surface through electrostatic interactions, with the functional groups acting as a Lewis base immobilizing the polysulfides [94,96,97]. There are also repulsive interactions that can diminish polysulfide migration [95]. Li et al. [102] hypothesized that there was an initial absorption of the polysulfides through hydrogen bonding, but the negatively charged functional groups cause electrostatic repulsion, preventing the diffusion of polysulfides from the cathode to the anode. Zhou et al. [103] ruled out the possibility of the cellulose separator trapping polysulfides, only reducing the shuttle effect through electrostatic repulsion. Diffusion of the polysulfides can also be physically blocked through the separator’s 3D structure given by the cellulose, as theorized by Zhang et al. [94] and Fang et al. [104], who used BC and PEI-modified BC, respectively, as separators.

Cellulose coatings have proven beneficial for improving the cycle performance of cells. Pan et al. [99] plasma treated a polyethylene (PE) separator to make it more hydrophilic. This treatment presented the advantage of depositing cellulose nanofibers (CNFs) without compromising the pore structure of the PE separators, due to its porosity being higher than that of the non-treated PE layer. The CNF coated on both sides of the separator provided a thermal shutdown mechanism to prevent thermal runway and was found useful in preventing lithium dendrite formation thanks to the controlled flux of lithium ions through the separator. No significant thermal shrinkage was found below 330 °C, which is the CNF decomposition temperature, demonstrating its high stability [99].

Recycled cellulose fibers (CFs) were also used to coat a polypropylene separator on the cathode side through vacuum filtration for use in Li-S cells. The CF-modified separator performed better electrochemically than the one without coating, with a longer lifespan and smaller capacity decay. While the addition of the coating layer increased the internal resistance of the battery initially, it decreased during cycling, while the internal resistance of the non-CF cell increased then stabilized. The battery with the CF coating cycled over 800 times with little capacity decay after the first two cycles, with a much more stable performance and smaller polarization in comparison to the non-CF separator [103].

Cellulose can also be used as an additive in the coating slurry. Shin et al. [98] used different amounts of nanocellulose in a ceramic particle coating layer. Nanocellulose spreads out ceramic Al_2_O_3_ particles, so the coating layer does not impair the pore structure, making it apt for electrolyte penetration. Symmetric lithium metal cells with coated separators exhibited lower overpotential and longer cycle life, with the best performance attributed to the 3:10 mass ratio in the Al_2_O_3_/NC composite; even though this was intended for an ion-Li cell, the strategy can be extended to Li-S batteries [96]. Li et al. [105] combined carbonized bacterial cellulose with TiO_2_ to modify a Celgard separator facing the sulfur cathode side. When carbonized, the network structure of bacterial cellulose is improved, which helps mitigate the shuttle by physically adsorbing the polysulfides in the cathode region.

Cellulose separators can be obtained through different methods. Yu and coworkers [106] tested a cellulose skin care mask as a separator in a Li-S battery. Its pore size was smaller than the Celgard 2500 pores, leading to a safer battery as larger pore sizes are more prone to internal short circuits by lithium dendrites. Polysulfide diffusion was also largely blocked by the separator, showing safe electrochemical performance for up to 1000 cycles at 0.2 C.

**Figure 11:**
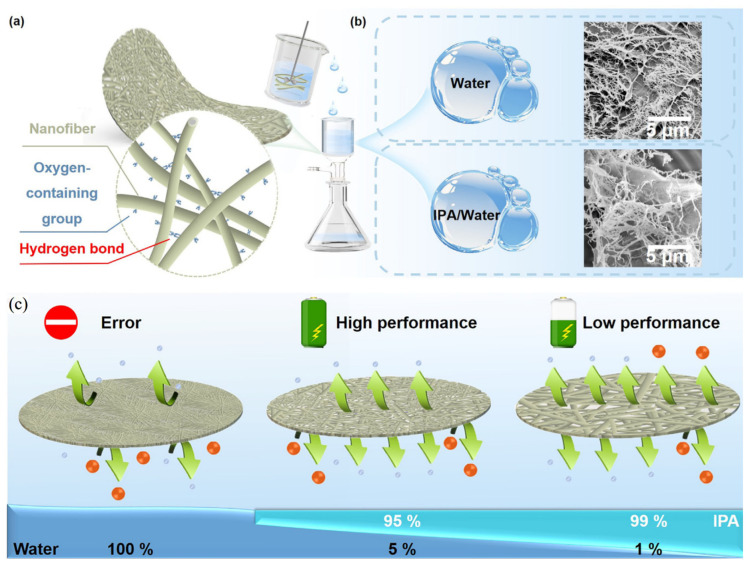
(**a**) Schematic illustration of the preparation of CNF separator and mechanism diagram of the interaction between CNFs (inset). (**b**) Cryo-SEM images of the CNFs in water (**top**) and IPA/water (**bottom**). (**c**) Schematic diagram of the relationship between suspension compositions, the pore structure of CNF separators and the electrochemical performance of LSBs. Reprinted with permission [95].

Vacuum filtration is a common facile method to prepare cellulose membranes. One of the main problems of this method is the resulting small pores. Li et al. [97] used a mixture of water and isopropanol (IPA) in different proportions as a cellulose nanofiber (CNF) dispersion medium to decrease its polarity and tune the separator’s pore size (Figure 11a), finding that increasing the amount of IPA increases the pore size. The separator prepared with a 99/1 IPA/water relation had a large pore size but showed a severe shuttle effect, while the one prepared in pure water did not allow for any ion migration (Figure 11b). Cycling performance improved when using a proper IPA/water (95/5) mixture compared to a PP separator due to the inhibition of the migration of polysulfides and the great wettability, delivering a higher discharge capacity and better cyclability.

Gou et al. [100] implemented another method to control the pore size in vacuum filtration, using polystyrene (PS) spheres with a diameter of 100 nm to enhance the porosity of the separator, which were then removed with a toluene bath. Different amounts of PS spheres were tested to obtain different pore sizes and porosities. Small and uniform pores regulate Li-ion flux; smaller pores lead to smaller dendrites according to experimental phenomena and numerical calculations. Greater porosity leads to the enhancement of wettability properties, but the separator’s mechanical properties are compromised as the porosity increases.

A cellulose vacuum-filtered separator with a carbon nanotube interlayer was prepared by Chien et al. [107] to be applied in Li-S batteries. The carbon interlayer increased the cell’s initial specific capacity; however, this study found that the shuttle effect became more severe with its presence. The interlayer slowed the diffusion of polysulfides, but their blocking was insufficient due to limited thickness. The cellulose separator and the cellulose separator with the interlayer both showed an improvement in the cyclability of the cell, given by the more homogeneous lithium deposition and slower electrolyte breakdown.

Park et al. [96] used the electrospinning method to fabricate a cellulose nanofiber-based separator, testing different separator thicknesses. The optimal amount of electrolyte was tested for each of them, and the separator with a 22 μm thickness required less electrolyte per mg of sulfur than the conventional Celgard separator, with their values being 14.2 and 21.2 μL·mgS^−1^, respectively, owing to polar functional groups and consequently better wettability and storage ability. Reducing the required electrolyte quantity also reduces the shuttle effect, as it decreases the polysulfide dissolution. Its unique 3D morphology and the oxygen-containing functional groups helped suppress Li dendritic growth. Wu and coworkers [108] modified an electrospun cellulose acetate (CA) membrane derived from recycled cigarette filters polymerizing a β-cyclodextrin and trimesoyl chloride (β-CD-TMC) in situ onto the CA membrane on the anode side of a Li-S cell. In addition to the modification with β-CD-TMC, the membrane was sputtered with carbon to form a 100 nm carbon layer in order to improve sulfur’s low electrical conductivity. The electrospun membrane had high porosity, through which polysulfides can pass, so the addition of the β-CD thin layer physically blocked the diffusion of long-chain polysulfides through its structure, acting as a sieve film, resulting in high initial discharge capacity of 1378.24 mAh g^−1^ and long-term cycling stability of 863.78 mAh g^−1^ after 1000 cycles at 0.2 C. The unmodified CA separator showed an inferior electrochemical performance in comparison to the one with the β-CD. These β-CD particles showed a “funnel effect”, promoting the transport of Li ions as it generates a differential ionic fluid pressure on both sides. Moreover, TMC reacts to form ester groups, which chemically anchor polysulfides.

Regenerated cellulose films were formed through the solubilization and later regeneration of cellulose with a nonsolvent and modified with polydopamine via immersion to improve its mechanical strength, wettability, and thermal stability by Fang et al. [101]. The high mechanical properties, good wettability, and uniform pore structure of the separator inhibited dendrite growth on the lithium metal anode, improving the Li/Li cell performance.

**Figure 12:**
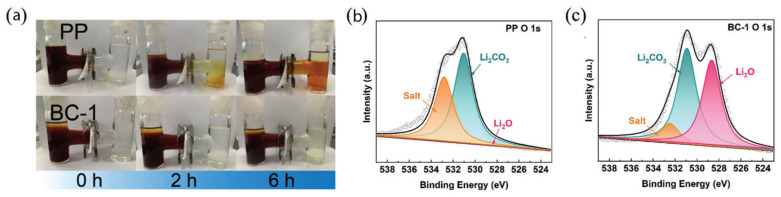
(**a**) Photos of Li_2_S_6_ diffusion across PP and BC separator clamped in H-type cell. XPS spectrums of O 1s high-resolution of cycled lithium anode surface in symmetric cell with (**b**) PP or (**c**) BC-1. Reprinted with permission [94].

Bacterial cellulose (BC) has been used in various ways for the fabrication of separators. Zhang et al. [94] tested bacterial cellulose aerogels with different thicknesses in Li-S batteries. The BC separator presented strong mechanical properties and uniform dispersion of lithium ions, inhibiting the growth of lithium dendrites and inhibiting the shuttle effect. The formation of LiO_2_ from the reaction between hydroxyl groups of the surface of the BC separator and the lithium anode was detected through XPS measurements (Figure 12). LiO_2_ is an electronic insulator that forms a passivation layer on the surface of the lithium anode, preventing fresh lithium deposition. Lesser amounts of oxide were found on the anode of the battery with the PP separator, leading to more dendrite growth. The BC separator with the best cycling stability had a thickness of 3 mm (130 μm inside the battery), balancing the captured polysulfides and ionic resistance.

Bacterial cellulose can also be oxidized, turning its hydroxyl groups into carboxylate groups. Li et al. [95] subjected BC pellicles to a (2,2,6,6-Tetramethylpiperidin-1-yl)oxyl (TEMPO) oxidation process and compared pristine and oxidized BC separators. The oxidation process separates the cellulose fibers into thinner nanofibers as it weakens the hydrogen bonds. This helps with the formation of dispersion, providing a more uniform membrane during the vacuum filtration. The oxidation improved the ionic conductivity, electrolyte uptake capability, and thermal stability in comparison to the pristine BC separator. It also reduces the cell’s self-discharge rate, as it prevents the diffusion of polysulfides further than the pristine BC separator and conventional Celgard separator [102]. The same process for the TEMPO oxidation of BC was used with the addition of SiO_2_ through in situ nucleation to obtain a BC-SiO_2_ composite film, resulting in a uniform coating. SiO_2_ reacts with hydroxyl groups on the surface, preserving the carbonyl groups that serve as another barrier for the polysulfide diffusion [95].

**Figure 13:**
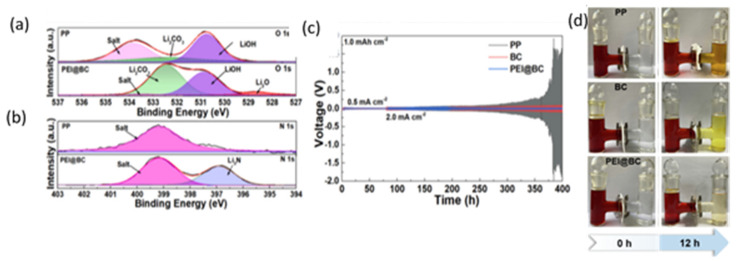
(**a**) O 1s and (**b**) N 1s high-resolution XPS spectrum of SEI layer after 250 s etching of PP and PEI@BC separator, respectively. (**c**) Galvanostatic cycling voltage profiles of Li/Li symmetrical cells assembled with different separators with a capacity of 1 mAh/cm^2^ (**d**) Visualized polysulfide diffusion test of these three separators using the H-shaped cell. (**c**) Optimized molecular models showing the interactions between PP, BC, PEI, PEI@BC. Reprinted with permission [95].

Fang et al. [104] fabricated a BC membrane modified with polyethyleneimine (PEI) through the impregnation method due to hydrogen bonding. The PEI modification led to smaller pore size as the BC nanofibers became more compact, but the grafting process did not change the thickness of the separator as other coating processes do. The intertwined structure of the BC and polar groups inhibited polysulfide diffusion. DFT simulations showed a strong interaction between Li_2_S_4_ and the PEI@BC separator due to the amino-rich PEI. Through XPS analysis, the presence of Li_3_N, one of the fastest Li-ion conductors, was detected only in the PEI@BC separator when compared to the BC and PP separators (Figure 13). Li_3_N and Li_2_O from the reaction of BC form a stable and ionic conductive SEI and inhibit the growth of lithium dendrites, exhibiting excellent cyclic stability, with a continuous plating/striping for more than 820 h with an overpotential of ~ 40 mV at 2 mA cm^−2^. The presence of the polar amino group restrains the polysulfide migration via chemosorption (Figure 13), achieving high capacity and great electrochemical performance in Li-S batteries.

### 4.3. Cellulose-Based Cathode Materials for Lithium–Sulphur Batteries

Li and co-workers [109] demonstrated that a carbon nanofiber aerogel (CNFA) obtained by pyrolysis of BC in an inert atmosphere exhibited extremely low density and excellent performance as a sulfur cathode, with an initial capacity of 1360 mAh g^−1^ at 0.2 C, as well as an excellent stability for 200 cycles. The porous structure of the CNFA allows for uniform sulfur distribution and high electrolyte absorption, improving its electrochemical performance, eliminating the need for current collectors, binders, or additives.

**Figure 14:**
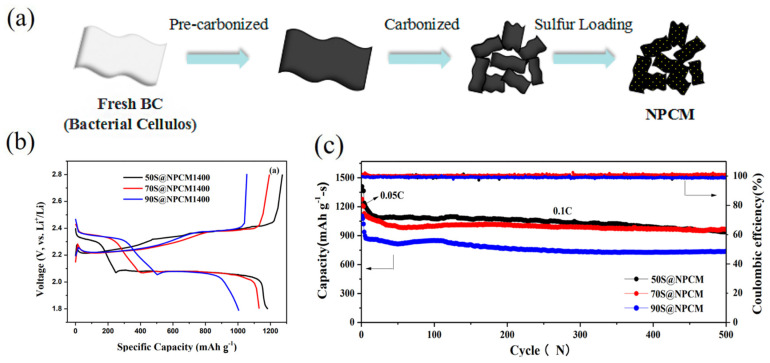
(**a**) Schematic illustration for NMCs and S@NMC composite fabrication. (**b**) Initial three charge–discharge profiles at a rate of 0.1 C of S@NMC composite cathode. (**c**) Cycling performance of 50S@NMC1400, 70S@NMC1400 and 90S@NMC1400. Reprinted with permission [110].

On the other hand, Quan et al. [110] developed microporous, nitrogen-doped carbon materials from BC by pyrolysis and activation with KOH (NMC). These materials exhibited high surface area and optimal electrical conductivity, which increases both the sulfur loading and the suppression of polysulfide diffusion. Cathodes based on these materials showed an initial specific capacity of 1267 mAh g^−1^ at 0.1 C and a retention of 995 mAh g^−1^ after 500 cycles, with a capacity attenuation of 0.03% per cycle. Nitrogen doping increased polysulfide adsorption and electronic conductivity, producing an effective matrix for long-life batteries (Figure 14).

Huang and co-authors [111] focused their research on refining the sulfur content in the cathode and evaluating its long-term performance. They synthesized a three-dimensional carbonaceous aerogel derived from BC as a flexible structure for its application in the cathode. After carbonization, the BC forms an interconnected network of nanofibers with high electrical conductivity and mechanical stability. The system with a high sulfur content (81 wt.%) achieved a specific capacity of 1134 mAh g^−1^ at a current density of 200 mAh g^−1^, reflecting its high energy storage capacity, while maintaining remarkable stability over prolonged cycling, with reversible capacities of approximately 800 mAh g^−1^ after 150 cycles. In long-term tests at a higher current density (400 mAg^−1^), the electrode retained a capacity of 700 mAh g^−1^ after more than 400 cycles, with an average Coulombic efficiency of 98.3%. Charge/discharge profiles revealed stable voltage plateaus throughout the 400 cycles, evidencing good reversibility and consistent performance.

Li et al. [112] studied the effectiveness of aerogels by testing carbon nanowires (CNRs) doped with nitrogen and oxygen, obtained through pyrolysis of BC. These CNRs were used to develop a sulfur cathode with a loading of 6.4 mg cm^−2^ and a specific capacity of 943 mAh g^−1^. This high capacity is attributed to the porous structure of the CNR and its ability to accommodate a large amount of sulfur.

Research conducted by Bharti et al. [113] addresses the use of BC as a separator and, once carbonized (CBC), as a cathode substrate. Its three-dimensional nanofiber structure strengthens the mechanical properties and optimizes the electrolyte distribution, enhancing its effectiveness. The CBC@BC design achieves an initial reversible capacity of 849 mAh g^−1^ at 1.0 C, maintaining 82% capacity after 500 cycles, with a low degradation rate (0.036% per cycle). The inclusion of Li_2_S_6_ catholyte ensures high sulfur loading and improves the distribution of active materials, raising the overall performance.

These studies are considered to be of great interest as they highlight the ability of BC-derived materials to offer significant improvements in electrical conductivity, polysulphide adsorption and long-term stability in Li-S batteries. In general, it can be observed that the common method employed by these researchers to obtain carbonaceous materials at the cathode is the application of pyrolysis at low and high temperatures.

Cellulose fiber from wood and cotton has also been used as a cathode material in Li-S batteries. Li et al. [114] fabricated nanofibrous activated carbon from common filter paper, showing a large surface area that, when impregnated with sulfur, resulted in a composite with an initial discharge capacity of 1393 mAh g^−1^ and 576 mAh g^−1^ after 100 cycles, thanks to its three-dimensional network structure that limits polysulphide dissolution and accommodates the volume expansion of sulfur.

Lu Li and co-workers [115] used electrospinning to encapsulate sulfur in a carbon-cellulose core–shell structure, achieving an initial discharge capacity of more than 1200 mAh g^−1^ and a Coulombic efficiency of more than 99% after 300 cycles. Here, the cellulose acts as an outer layer, encapsulating the carbon/sulfur compound (CMK-3/S), and the process includes the use of solvents such as ionic liquid and surfactants. Na Li and co-authors [116] developed a self-supporting cathode with a multiscale lattice structure using pulp fibers and carbon nanotubes, showing an initial capacity of 1204.6 mAh g^−1^ with excellent cyclic stability without the need to use high temperatures relying on self-assembly techniques and papermaking-inspired processes to improve the structure and performance of the battery. This resulted in a capacity retention rate of 95.4% after 200 cycles at 0.2 C, with the final specific discharge capacity being 952 mAh g^−1^. Liu and co-authors [117] reported the fabrication of aerogels composed of cellulose nanofibers and MXene, obtaining an aerogel with superior performance, achieving a high capacity of 1573.7 mAh g^−1^ and a capacity retention of 96.3% after 200 cycles. These works show the potential of cellulose nanofiber-derived materials to address the challenges of conductivity and stability in Li-S batteries, offering innovative and efficient solutions.

Cellulose nanocrystals (CNCs), cellulose acetate (CA), and composite materials have also been applied as cathode materials in Li-S batteries. Sevilla et al. [118] used cellulose and cellulose acetate as precursors to synthesize highly micro–mesoporous carbon biochars with high surface areas above 2500 m^2^ g^−1^ and sulfur/carbon composites by chemical activation with sodium thiosulphate. The sulfur/carbon composites reached capacities up to 1000 mAh g-1 S during the initial discharge at 0.1 C and still above 500 mAh g^−1^ S at 2 C.

**Figure 15:**
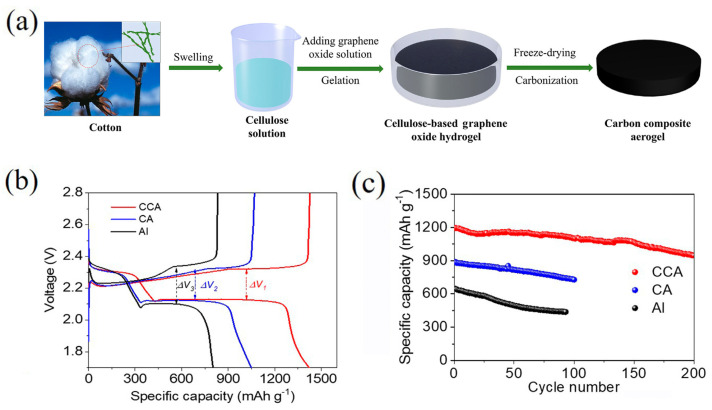
(**a**) Schematic illustration of the preparation technology of CCA; (**b**) charge–discharge voltage profiles for three different current collectors at a current density of 0.1 C (1 C = 1675 mAh g^−1^); and (**c**) cycling performance at 0.2 C. Reprinted with permission [120].

Mao et al. [119] synthesized self-standing 3D cellulose graphene-based aerogel with good flexibility, low density, and high porosity with an excellent electrolyte absorption, improving the charging and discharging efficiency of sulfur, showing high specific capacity of 945.79 mAh g^−1^ after 200 cycles at 0.2 C and improved electrochemical reaction kinetics. Cellulose-based materials can also be used as a binder in the cathode with great results. Yu et al. [120] presented an innovative approach involving the fabrication of a cellulose-based graphene aerogel with interconnected 3D structure combined with CeO_2_ to improve the reaction kinetics and electrochemical performance of LiSB, showing a remarkable advance in cyclic stability and overall capability (Figure 15).

Liu et al. [121] studied cellulose nanocrystals (CNCs) as a multifunctional binder for Li-S batteries. The low-cost, environmentally friendly, and hydrophilic CNCs not only firmly confine sulphur and polysulfide at the cathode but also prevent polysulfide transport to the Li anode by acting as a polysulfide plug in the separator. Theoretical calculations suggest that the electron-rich functional groups in the CNCs provide robust binding energies to the polysulfide. The results show that Li-S batteries with CNCs demonstrate outstanding cyclic performance even under conditions of high sulfur loading of 90 wt% (63 wt% at the cathode), high charge of 8.5 mg cm-g^−2^ and elevated temperature of 60 °C.

## 5. Conclusions and Perspectives

Cellulose-based materials have emerged as a versatile and sustainable solution to address the challenges faced by lithium–sulfur (Li-S) batteries, including lithium dendrite growth, the polysulfide shuttle effect, and poor sulfur utilization. Their unique properties, such as renewability, mechanical robustness, ionic conductivity, and the ability to be chemically modified, make them highly effective in various battery components.

For lithium metal protection, cellulose materials as coatings and composites provide enhanced interfacial stability, suppress dendrite formation, and promote uniform lithium deposition thanks to their many polar functional groups and strong compatibility with lithium. It improves ionic distribution in the SEI stability and formation and, thus, inhibits dendrite formation through more homogeneous lithium deposition and stripping. Consequently, the addition of cellulose in the lithium protection allows for the stabilization of the lithium metal anode, extending the cycle life of Li-S batteries and improving their safety.

As material in separators, cellulose-based materials offer excellent thermal and chemical stability, high ionic conductivity, and the ability to trap polysulfides, significantly reducing capacity fading and enhancing cycling stability. Cellulose separators have shown a positive impact on the decrease in both lithium dendrite growth and LiPS shuttling. More importantly, by engineering a separator with a uniform nanoscale pore distribution and functional chemical group distribution, the Li+ flux at the anode surface will improve that of conventional polyolefin separators, resulting in a more uniform lithium deposition.

Cathode material cellulose-derived carbon frameworks and functionalized cellulose enhance sulfur loading, improve electrical conductivity, and ensure structural integrity, leading to higher energy density and better long-term performance. The distribution of sulfur in the cathode is assisted by the pore structure of cellulosic materials. This homogeneous distribution not only improves electrical conductivity but also reduces the formation of polysulfides. As a result, cathodes with higher specific capacity, excellent cyclic stability, and extended lifetime are achieved. Cellulose serves as a porous carbon framework, enhancing the electrochemical performance and ensuring a better utilization of sulfur active material, contributing to a higher specific energy thanks to its light weight.

The high versatility of cellulose-based materials allows for developing advanced functionalized cellulose materials, such as those doped with conductive additives, catalytic species, or polar functional groups, which can further enhance lithium-ion kinetics, polysulfide adsorption, and redox reactions, which has not been exploited in energy storage materials to improve its chemical and physical properties. Different types of cellulose have the potential to offer unexplored benefits and functionalities. To our knowledge, hairy nanocellulose and its derivatives are yet to be used in the fabrication of electrodes or separators for lithium batteries. Tailoring the porosity and structure of cellulose membranes and carbon derivatives is important in order to optimize electrolyte compatibility and active material utilization. More research on the interaction between cellulose and lithium polysulfides is needed to better understand cellulose’s advantages and its working mechanism. Future research could focus on theoretical studies and in situ measurements during battery cycling.

It is also important to prioritize scalable and cost-effective methods for producing cellulose-based components, such as 3D printing, electrospinning, or green synthesis techniques, to facilitate commercialization. Exploring non-wood sources of cellulose, such as agricultural waste or bacterial cellulose, can further reduce production costs and environmental impact.

Cellulose is biodegradable and a greener alternative in comparison to other materials used in batteries, and yet many of the solvents and mechanical procedures used for its processing are not totally environmentally friendly. Conducting comprehensive lifecycle assessments to evaluate the environmental impact of cellulose-based materials in Li-S batteries is essential for validating their green credentials. Recycling and reusing cellulose-based components could further enhance the sustainability of Li-S battery systems.

Cellulose-based materials represent a promising pathway for overcoming the inherent challenges of Li-S batteries while promoting sustainability. With ongoing advancements in materials science, engineering, and electrochemistry, cellulose-based technologies are poised to play a pivotal role in enabling high-performance, eco-friendly, and commercially viable Li-S batteries for future energy storage needs.

## Figures and Tables

**Table 1:** Main properties of the different composite solid polymer electrolytes with cellulose applied to lithium metal batteries.

Type of Cellulose	Composite	Electrochemical Window [V]	Li Transfer Number	Li ionic Conductivity [Scm^−1^] [MPa]	Tensile Stenght [MPa]	Storage Modulus [MPa]	Ref.
Methyl 2-hydroxyethyl cellulose (HEMC)	PEO-LiTFSI-20HEMC	0–5.56	0.467	1.30 × 10^−4^ at 30 °C 1.68 × 10^−3^ at 60 °C	3.26	46.95	[74]
Bacterial cellulose (BC)	BC as a polymer filler incorporated with PEO in a LiTFSI based electrolyte	0–1.43	0.57	0.56 × 10^−4^ at 30 °C	4.43	76.7	[76]
Cellulose nanofibers (CNFs)	Comprised soft poly(ethylene oxide co-epichlorohydrin) (EO-co-EPI) with CNF	0–4	-	6 × 10^−5^ at 25 °C	-	64	[73]
Commercial membrane cellulose (CF)	Succinonitrile (SN) plasticizer in addition to zeolitic imidazolate frameworks (ZIFs) grown in situ on the CF skeleton, all into PEO	0–5	0.40	1.17 × 10^−4^ at 30 °C	18.7	295.85	[77]
Polyurethane-cellulose acetate (PCA)	Blend of PCA with mono-hydroxysiloxane-capped polyethylene oxide (PU-CA)	-	-	1.5 × 10^−5^ at 25 °C	-	-	[78]
Nano scale micro-fibrillated cellulose (MCF)	MCF fibers reinforced fully-solid methacrylic-based thermo-set polymer electrolyte membranes	0–4.7	-	0.1 × 10^−3^ at 50 °C	2.3	32	[80]
Robust cellulose nonwoven (RCNW)	Poly (ethylene oxide), poly (cyano acrylate), lithium bis(oxalate)borate with RCNW	0–4.6	-	3 × 10^−4^ at 60 °C	43	-	[81]
Methyl cellulose (MC)	Blending of LiClO_4_, MC and Pegylated Polyoctahedralsilsesquioxane	1.5–4.2	0.25	1.6 × 10^−5^ at 30 °C and 1.1 × 10^−5^ at 0 °C	-	32	[82]
Ethyl cellulose (EC)	PEO, LiTFSI and EC mixture	0–4	0.9	0.52 × 10^−4^ at 70 °C	-	-	[83]
Carboxymethyl cellulose (CMC)	CMC-lithium perchlorate and citric acid	0–2.15	-	1.24 × 10^−7^	-	-	[84]
Methyl cellulose (MC)	Blend of MC with LiPF_6_-based organic electrolyte	0–4.8	0.48	0.7 × 10^−3^ at 30 °C	5.8	-	[85]
Cellulose acetate (CA)	Mixture of CA with inorganic NASICO-type electrolyte Li_1.3_Al_0.3_Ti_1.7_(PO_4_)_3_	0–4.6	0.85	6.17 × 10^−4^ at 30 °C	1.3	-	[86]

## Data Availability

Data sharing is not applicable.
